# Complete sequencing of the mitochondrial genome of tea plant *Camellia sinensis* cv. ‘Baihaozao’: multichromosomal structure, phylogenetic relationships, and adaptive evolutionary analysis

**DOI:** 10.3389/fpls.2025.1604404

**Published:** 2025-06-13

**Authors:** Zhiyin Chen, Zixu Wang, Wei Zhou, Sijie Liu, Yuxin Xiao, Yihui Gong

**Affiliations:** ^1^ College of Agriculture and Biotechnology, Hunan University of Humanities, Science and Technology, Loudi, Hunan, China; ^2^ Key Laboratory of Characteristic Agricultural Resource Development and Quality Safety Control in Hunan Province, Hunan University of Humanities, Science and Technology, Loudi, Hunan, China; ^3^ Innovation and Entrepreneurship Education Center for Horticultural Production and Processing in Hunan Province, Hunan University of Humanities, Science and Technology, Loudi, Hunan, China; ^4^ Hunan University of Humanities, Science and Technology, Hunan Provincial Innovation and Entrepreneurship Demonstration Base, Loudi, Hunan, China

**Keywords:** *Camellia sinensis* cv. ‘Baihaozao’, mitochondrial genome, multichromosomal structure, adaptive evolution, phylogenetic relationships

## Abstract

**Introduction:**

This study reports for the first time the complete sequence characteristics of the mitochondrial genome of the tea plant cultivar Camellia sinensis cv. ‘Baihaozao’. It systematically unveils its multi-chromosomal structure, RNA editing patterns, and adaptive evolutionary mechanisms, providing critical theoretical insights into the structural complexity and evolutionary mechanisms of the tea plant mitochondrial genome.

**Methods:**

The mitochondrial genome was fully analyzed using genome sequencing and annotation techniques. RNA editing sites were predicted to evaluate editing patterns. Codon usage bias analysis was conducted to identify high-frequency codons. Repeat sequence analysis was used to characterize dispersed and tandem repeats. Adaptive evolutionary analysis, based on Ka/Ks ratios, was performed to investigate gene selection pressures.

**Results:**

The mitochondrial genome consists of 11 linear chromosomes, with a total length of 909,843 bp and a GC content of 45.62%. A total of 73 functional genes were annotated, among which 14 variable genes (e.g., ribosomal protein coding genes) retain intact functions without pseudogenization, which is rare among Theaceae plants. RNA editing site prediction revealed significant spatial heterogeneity, with the cox1 gene being a hotspot containing 19 editing sites. Approximately 58.49% of editing events were concentrated on the second base of codons, and 48.61% of the sites resulted in amino acid changes from hydrophilic to hydrophobic. Codon usage bias analysis showed significant enrichment of high-frequency codons, including UUU (phenylalanine), AUU (isoleucine), and UUC (phenylalanine). The genome’s repeat sequences were predominantly dispersed repeats (70.6%), with forward and palindromic repeats of 30–40 bp being dominant. Tandem repeats exhibited significant distribution heterogeneity among chromosomes. Adaptive evolution analysis showed that most PCGs (protein-coding genes) had Ka/Ks ratios below 1 (ranging from 0.07 to 0.78), with the atp9 gene showing the lowest ratio (0.07), while the mttB gene exhibited a significantly higher Ka/Ks ratio of 3.48. Additionally, 1.62% of the mitochondrial genome sequence was homologous to the chloroplast genome, carrying 26 complete functional genes, including 15 tRNA and 2 rRNA genes.

**Discussion:**

Codon usage bias may be related to mutation pressure due to the high AT content of the genome or reflect adaptive selection pressures for translational efficiency. The Ka/Ks results align with the widespread purifying selection observed in mitochondrial genomes, while the high Ka/Ks ratio of the mttB gene suggests it might be under positive selection to adapt to environmental pressures. The evolutionary evidence of inter-organelle gene transfer highlights the homologous sequences between mitochondria and chloroplasts. Overall, these findings systematically elucidate the adaptive evolutionary mechanisms and functional regulation of the tea plant mitochondrial genome.

## 1 Introduction

The mitochondrial genome of plants, as a unique vector of maternal inheritance, holds significant scientific value in studies of phylogenetics and genetic diversity due to its dynamic recombination characteristics and adaptive evolutionary mechanisms (Lu et al., 2022; Li et al., 2023a). In recent years, breakthroughs in third-generation sequencing technologies have enabled research on the mitochondrial genomes of *Camellia* species, revealing key features such as gene recombination, dynamic equilibrium of repetitive sequences, and inter-organellar gene transfer (Lu et al., 2022; Li et al., 2023a). However, existing studies predominantly focus on cultivated varieties (e.g., *C. sinensis* var. *assamica*) and oilseed camellia (*C. gigantocarpa*) (Lu et al., 2022; Li et al., 2023a), while structural analyses of mitochondrial genomes in unique varieties of both economic and ecological significance—such as the early-germinating and stress-resistant tea cultivar *Camellia sinensis* cv. ‘Baihaozao’—remain unexplored. Traditional mitochondrial genome studies have largely been based on a single-chromosome model (Kozik et al., 2019). However, the recently revealed multichromosomal structures in species such as cucumber (*Cucumis sativus*) and poplar (*Populus simoni*i) (Alverson et al., 2011; Bi et al., 2022) suggest that the mitochondrial genome of Camellia may exhibit greater structural diversity, which urgently requires investigation using advanced technological approaches.

Current research reveals that the mitochondrial genome of tea plants exhibits significant recombination polymorphism: the cultivated variety Duntsa (*C. sinensis* var. *assamica* cv. *Duntsa*) contains 18 homologous recombination regions, while different varieties (*Camellia sinensis* var. *assamica* OL, CSAOL and *Camellia sinensis* var. *assamica* OM, CSAOM) show a 44% difference in the distribution of repetitive sequences and the number of introns (Li et al., 2023a). Notably, the codon usage bias of the *atp9* gene is driven by mutational pressure, whereas other genes are primarily influenced by natural selection (Li et al., 2023a). Additionally, the pseudogenization of the rps16 gene and mitochondrial-chloroplast gene transfer events (e.g., homologous sequences in the inverted repeat (IR) region accounting for over 90%) (Zhao et al., 2018) collectively outline the dynamic evolutionary landscape of the tea plant mitochondrial genome. However, these findings are based on the assumption of a single-chromosome model, overlooking critical issues such as the potential differences in allelic recombination frequency (Alverson et al., 2011) and replication autonomy (Bi et al., 2022) that may arise from multi-chromosomal structures. Of particular interest is the recent genome sequencing of *Lingyun Baihao Tea*, which reveals that wild germplasm differs significantly from cultivated varieties in morphological traits such as leaf area, serration density, and trichome coverage (He et al., 2023). This provides important insights into the relationship between mitochondrial genome structural variation and phenotypic plasticity.

As a unique early-maturing and high-quality germplasm native to China, *Camellia sinensis* cv. ‘Baihaozao’ serves as an ideal model for elucidating the adaptive evolution of mitochondrial genomes due to its distinctive stress-resistant metabolic pathways and rapid environmental response characteristics. This study employs an innovative hybrid sequencing strategy combining Illumina second-generation and third-generation sequencing technologies, overcoming the technical limitations of traditional circular genome assembly (Kozik et al., 2019), and successfully achieves the complete sequencing and assembly of a multi-chromosomal mitochondrial genome in *Camellia*. By integrating comparative genomics and adaptive evolution analyses, this study aims to elucidate: (1) the patterns of gene recombination under multi-chromosomal architectures and their impact on phenotypic plasticity; (2) the evolutionary relationship between *rps16* gene pseudogenization and inter-organellar interactions; and (3) positive selection signals associated with stress-resistance traits and their molecular regulatory networks. These findings will fill the research gap in the mitochondrial genome of *Camellia sinensis* cv. ‘Baihaozao’ and provide a theoretical foundation for crop genetic improvement.

## 2 Materials and methods

### 2.1 Preparation of mitochondrial genome deoxyribonucleic acid

**Figure 1:**
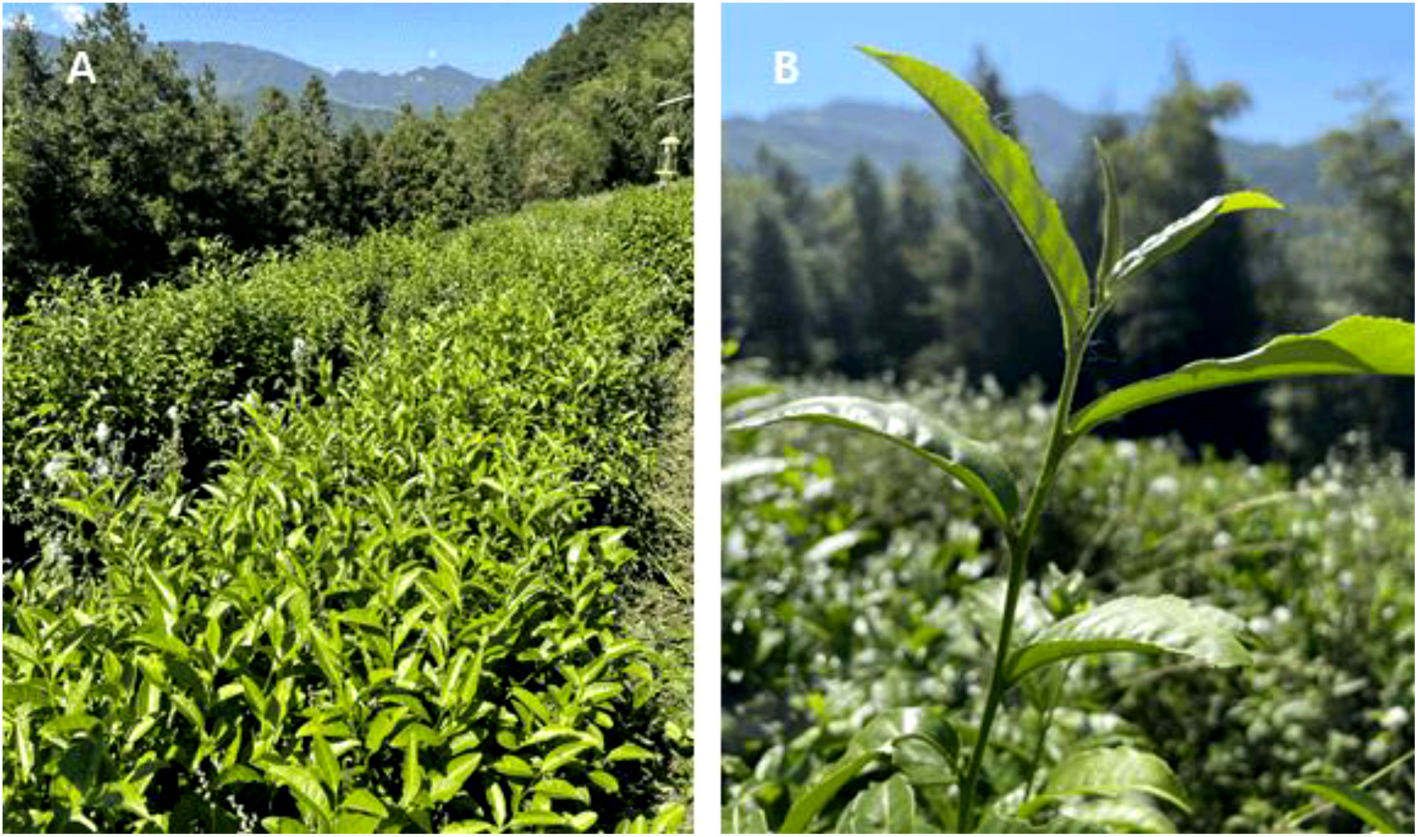
The planted field **(A)** and single tea plant of *Camellia sinensis* cv. ‘Baihaozao’ **(B)**.

The experimental materials were obtained from tender shoots of tea plants (*Camellia sinensis* cv. ‘Baihaozao’) cultivated at Taohuayuan Modern Agriculture Development Co., Ltd., Xinhua County, Hunan Province, China (27°47′N, 110°51′E) (**Figure 1**). Total DNA was extracted using the TIANGEN Plant Genomic DNA Extraction Kit (DP305). Dual quality control was performed to ensure DNA integrity (fragment length >30 kb) and purity using a spectrophotometer (A260/A280 = 1.8–2.0, A260/A230 ≥ 2.0) and 1% agarose gel electrophoresis (0.8% agarose gel, 5 V/cm, 30 min) (Cui et al., 2024; Zhang et al., 2024a).

### 2.2 Multi-omics sequencing strategy

A hybrid sequencing library was constructed using Illumina NovaSeq 6000 (PE150 mode) and Oxford Nanopore PromethION (R10.4.1 flow cell). The construction of the second-generation library included standardized procedures such as ultrasonic fragmentation of 350 bp insert fragments, end repair using NEBNext Ultra II, A-tailing with Klenow polymerase, adapter ligation using T4 DNA ligase, and selection of target fragments with AMPure XP magnetic beads. The third-generation library was prepared using the Ligation Sequencing Kit (SQK-LSK114), followed by large-fragment selection using BluePippin (>30 kb selection window) (Shin et al., 2019; Cui et al., 2024).

Sequencing Data Preprocessing: Raw second-generation sequencing data were filtered using fastp (v0.20.0) with the following parameters: -q 5 -n 5 -l 50. Nanopore sequencing data underwent basecalling using Guppy (v6.4.2), removal of chimeric adapters using Porechop (v0.2.4), and error correction of long reads using LoRDEC (v0.6) (Shin et al., 2019; Yang and Duan, 2024). As a result, 20 Gb of second-generation clean data (Q30 > 90%) and third-generation data with 50× genome coverage (N50 > 30 kb) were obtained.

### 2.3 Multi-chromosomal assembly and validation

Using an adaptive k-mer weighting algorithm, *de novo* assembly of third-generation sequencing data was performed with Canu (v2.2) (parameters: genomeSize=600 k, corMhapSensitivity=high, corMinCoverage=50). Contigs were aligned to the NCBI reference genome (NC_037880.1) using Minimap2 (v2.24), and mitochondrial homologous sequences were identified via BlastN (e-value=1e-5) (Li, 2018; Yang and Duan, 2024).

Assembly Optimization Workflow: Iterative correction of the assembly was conducted using Pilon (v1.24) with second-generation data (3 rounds); Hybrid data polishing was performed using NextPolish (v1.3.1); linear structures were validated using Circlator (v1.5.5) and confirmed by reverse polymerase chain reaction (PCR) amplification (PrimeSTAR GXL DNA polymerase) and Sanger sequencing at the junction sites (Shin et al., 2019; Schiavone et al., 2022). The final assembly yielded a complete mitochondrial genome with a multi-chromosomal structure. Evaluation using QUAST (v5.2.0) demonstrated high assembly continuity (N50 >100 kb) and completeness (BUSCO > 95%), meeting reference genome standards (Schiavone et al., 2022; Yang and Duan, 2024).

### 2.4 Annotation of mitochondrial gene structure

The annotation of protein-coding genes and rRNA was performed using the BLAST algorithm (gapped BLAST and PSI-BLAST) through multiple sequence alignments with published plant mitochondrial reference genomes. Manual curation was conducted to ensure annotation accuracy. tRNA genes were systematically identified using tRNAscan-SE software, which employs a covariance model-based algorithm to efficiently detect secondary structural features of transfer RNAs, significantly reducing false-positive rates. Open reading frames were predicted using the national center for biotechnology information (NCBI) Open Reading Frame Finder with a minimum length threshold of 102 bp, and the results were manually corrected to exclude non-functional fragments (Tatusova et al., 2016). The final annotation results were visualized using the OGDRAW tool to generate a genomic circular map and submitted to the NCBI database in tbl format, adhering to standard mitochondrial genome annotation protocols (Tatusova et al., 2016; Li et al., 2023a).

### 2.5 Analysis of mitochondrial genome repeats

Dispersed repeat sequences were identified using vmatch (v2.3.0) in combination with Perl scripts. The parameters were set to a minimum length of 30 bp and a Hamming distance of 3, covering four types of repeats: direct, palindromic, reverse, and complementary. Simple sequence repeats (SSRs) were identified using the MISA software, which utilizes a statistical model based on repeat unit counts to systematically detect microsatellite loci, providing marker resources for subsequent population genetics studies (Li et al., 2023a).

### 2.6 Phylogenetic analysis

The mitochondrial genome sequences of 26 representative plant species from five different families were downloaded from the NCBI database. Phylogenetic trees were constructed using the maximum likelihood and neighbor-joining methods in MEGA 7.0 software. Bootstrap resampling was performed 1000 times to evaluate node support. The analysis was based on nucleotide substitution models (e.g., Kimura 2-parameter) to ensure the statistical reliability of the tree topology (Li et al., 2023a).

### 2.7 Ka/Ks analysis

Pairwise comparisons of homologous genes were conducted using MAFFT (v7.310) for multiple sequence alignment, followed by calculations of non-synonymous substitution rate (Ka) and synonymous substitution rate (Ks) using KaKs_Calculator v2.0. The MLWL algorithm was employed to optimize the evaluation of evolutionary pressure. The results were presented as box plots, revealing the functional divergence of different genes during adaptive evolution (Mao et al., 2005; Li et al., 2023a).

### 2.8 RNA editing sites and variation analysis of the mitochondrial genome

RNA editing sites were predicted using the PREPACT online platform, which employs an algorithm based on the conservation of plant mitochondrial RNA editing sites to identify C-to-U transition sites (Jühling et al., 2012). Nucleotide diversity (π value) was calculated using DNAsp v5 after global alignment with MAFFT (v7.427, –auto mode), reflecting the degree of sequence variation among species (Li et al., 2023a). Homologous regions between the chloroplast and mitochondrial genomes were identified using BLAST (similarity ≥70%, E-value ≤10^-5^) and visualized with Circos (v0.69-5) to depict genome-wide synteny relationships (Li et al., 2023a). Synteny analysis was performed using nucmer (v4.0.0beta2, –maxmatch parameter), and a dot-plot was generated to reveal genome rearrangement events (Li et al., 2023a).

## 3 Results

### 3.1 Multi-chromosomal structure of the mitochondrial genome in *Camellia sinensis* cv. ‘Baihaozao’

In this study, the mitochondrial genome of *Camellia sinensis* cv. ‘Baihaozao’ was sequenced using the Illumina NovaSeq 6000 high-throughput sequencing platform. Quality analysis of the sequencing data showed that the Q20 (the proportion of bases with a quality score ≥ 20) and Q30 (the proportion of bases with a quality score ≥ 30) reached 97.00% and 92.27%, respectively, meeting the typical performance metrics of the platform, where Q20 generally exceeds 97% and Q30 surpasses 92% in transcriptome sequencing. After quality control of the sequencing data, a total of 16.14 Gb (16,140,581,400 bp) of clean data was obtained, which falls within the theoretical throughput range of a single run on the Illumina platform. Detailed data information is provided in **Supplementary Table 1**.

**Figure 2:**
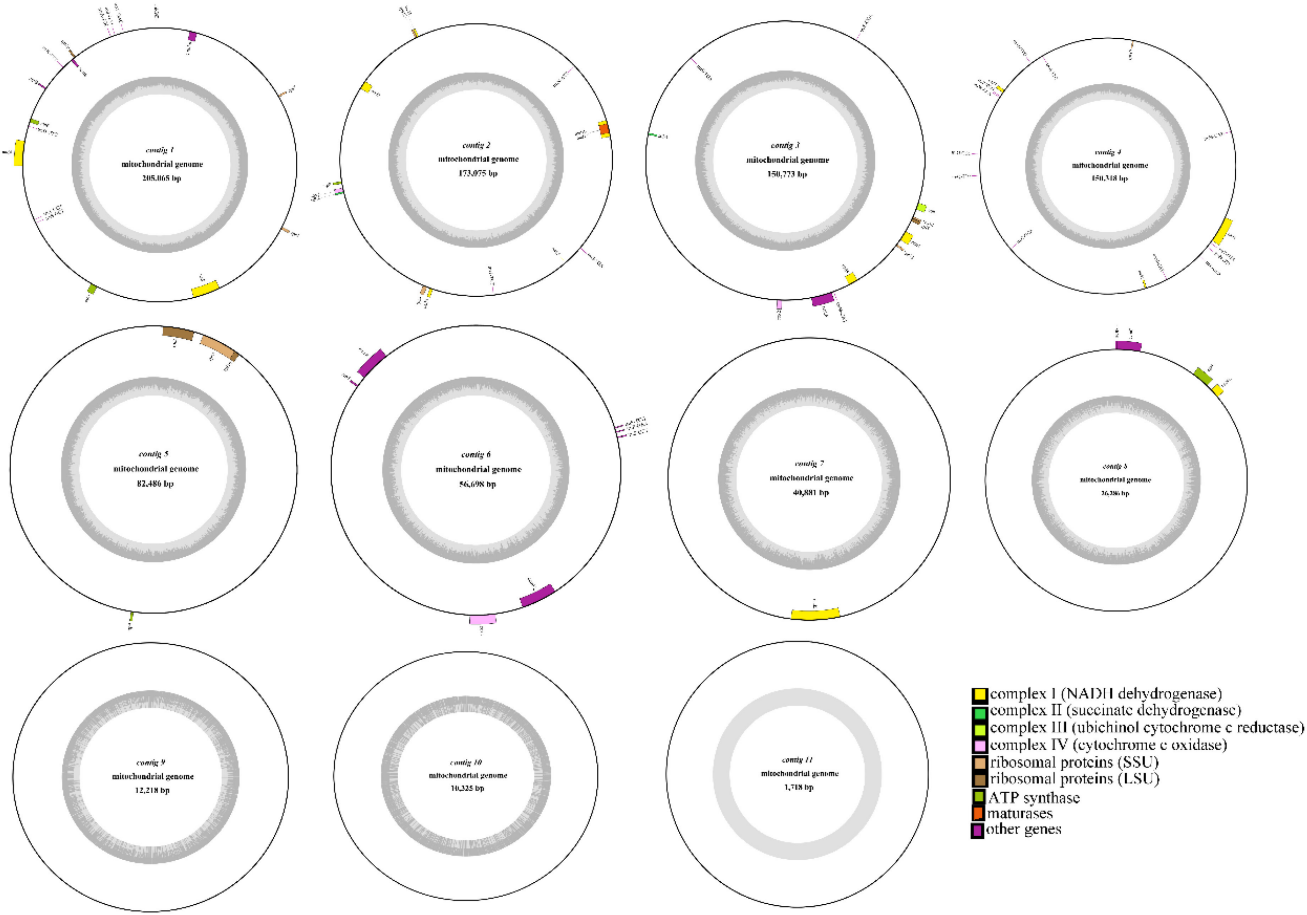
Mitochondrial genome map. The mitochondrial genome consists of 11 linear chromosomes. In the linear diagram, forward-strand genes are located on the outer side of the circle, while reverse-strand genes are on the inner side. The inner gray circle represents GC content.

This study conducted deep sequencing and assembly of the mitochondrial genome of the tea cultivar *Camellia sinensis* cv. ‘Baihaozao’. The complete mitochondrial genome sequence has been submitted to GenBank (accession numbers: PV340641–PV340651). The results revealed that the mitochondrial genome is composed of 11 linear chromosomes (**Figure 2**; **Supplementary Table 2**), with a total length of 909,843 bp (individual chromosome lengths ranging from 1,718 to 205,065 bp) and a GC content of 46% (**Supplementary Table 3**). This multi-chromosomal structure is consistent with the characteristic mitochondrial genome organization commonly observed in Theaceae plants (Li et al., 2023a), but the total length is significantly shorter than that of *C. sinensis* var. *assamica* cv. ‘Duntsa’ (1.08 Mb), indicating notable intraspecific length polymorphism in tea mitochondrial genomes.

Genome annotation identified a total of 73 functional genes, including 38 protein-coding genes, 30 tRNA genes, 3 rRNA genes, and 2 pseudogenes (**Table 1**; **Supplementary Table 4**). Among these, the GC content of protein-coding genes (43%) was significantly lower than that of tRNA genes (50%) and rRNA genes (52%), which may be attributed to differential selective pressures acting on functional elements (Li et al., 2023a). The core gene set includes 24 conserved genes, covering adenosine triphosphate (ATP) synthase subunits (*atp1/4/6/8/9*), nicotinamide adenine dinucleotide (NADH) dehydrogenase subunits (*nad1-7/9*), cytochrome c synthesis-related genes (*ccmB/C/Fc/Fn*), and cytochrome oxidase subunits (*cox1-3*). This gene composition aligns closely with the conserved features of mitochondrial genomes in Theaceae plants (Li et al., 2023a). Notably, the 14 variable genes include those encoding ribosomal large subunit proteins (*rpl2/5/10/16*) and small subunit proteins (*rps1/3-4/7/12-14/19*). These genes are frequently lost or pseudogenized in some Theaceae plants (Li et al., 2023a), yet they remain fully functional in *Camellia sinensis* cv. ‘Baihaozao’. This suggests that the mitochondrial genome of *Camellia sinensis* cv. ‘Baihaozao’ exhibits a relatively high degree of evolutionary conservation.

**Table 1:** Functional classification of genes in the *Camellia sinensis* cv. ‘Baihaozao’ mitochondrial genome.

Group of genes	Gene name
ATP synthase	*atp1 atp4 atp6 atp8 atp9*
Cytohrome c biogenesis	ccmB ccmC ccmFc* ccmFn
Ubichinol cytochrome c reductase	*cob*
Cytochrome c oxidase	*cox1 cox2 cox3*
Maturases	*matR*
Transport membrane protein	*mttB*
NADH dehydrogenase	nad1**** nad2**** nad3 nad4** nad4L nad5**** nad6 nad7**** nad9
Ribosomal proteins (LSU)	rpl10 rpl16 rpl2* rpl5
Ribosomal proteins (SSU)	#rps19 rps1* rps12 rps13 rps14 rps19 rps3* rps4 rps7
Succinate dehydrogenase	*#sdh3 sdh4*
Ribosomal RNAs	*rrn18 rrn26 rrn5*
Transfer RNAs	trnA-TGC* trnC-GCA(2) trnD-GTC trnE-TTC trnF-GAA trnG-GCC trnH-GTG trnI-GAT*(2) trnK-TTT trnM-CAT(4) trnN-ATT trnN-GTT trnP-TGG(2) trnQ-TTG trnS-CGA trnS-GCT(2) trnS-TGA trnS-TGA* trnT-GGT* trnT-TGT* trnV-GAC trnW-CCA trnY-GTA
other	

*: intron number; #Gene: Pseudo gene; Gene (2): Number of copies of multi-copy genes.

Gene structure analysis revealed that *trnM-CAT* has four copies, while *trnC-GCA* and *trnI-GAT* are present in two copies. The distribution of introns showed significant heterogeneity: *nad4* contains two introns, while *nad1/2/5/7* each contain four introns, consistent with the intron expansion trend observed in the mitochondrial genomes of Theaceae plants (Li et al., 2023a). Notably, the *sdh3* and *rps19* genes each contain one pseudogene, which may be associated with gene fragmentation during mitochondrial genome recombination (Li et al., 2023a). Such structural variations might play important roles in the adaptive evolution of Camellia species. For example, the retention of the *sdh4* gene may contribute to the assembly of the succinate dehydrogenase complex, potentially influencing energy metabolism pathways (Wei et al., 2017).

This study is the first to systematically reveal the multi-chromosomal structural characteristics of the mitochondrial genome in *Camellia sinensis* cv. ‘Baihaozao’. The gene composition shows significant differences compared to closely related species, such as *C. sinensis* var. *assamica* (Li et al., 2023a). A comparison with the published chloroplast genome data revealed that the GC content of the mitochondrial genome (46%) is significantly higher than that of the chloroplast genome (37%). This difference may be attributed to the varying selective pressures acting on the genomes of different organelles (Li et al., 2023a).

### 3.2 Prediction of RNA editing sites

**Figure 3:**
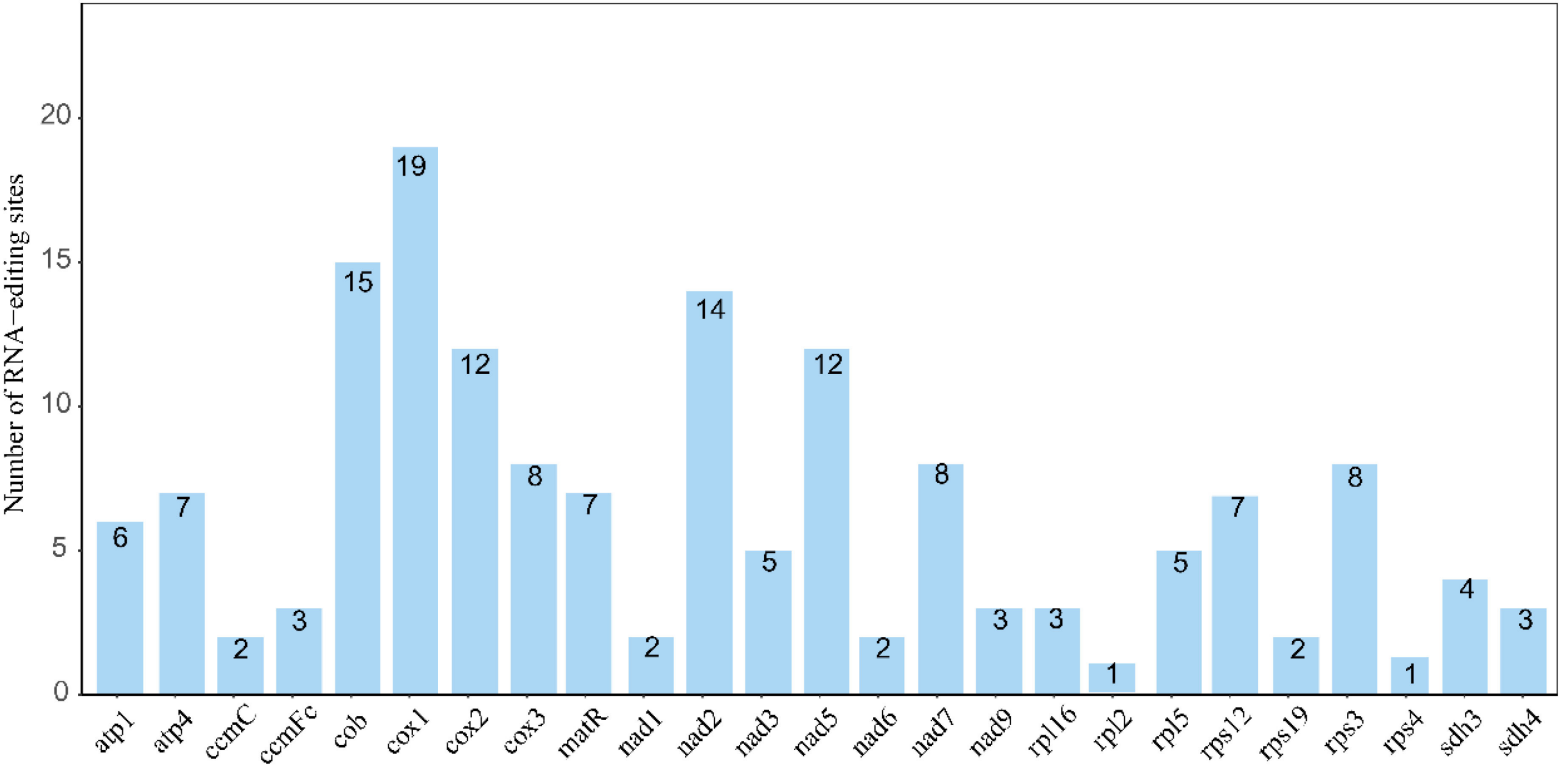
Statistics of RNA editing site numbers for each gene. The horizontal axis represents genes, and the vertical axis represents the number of RNA editing sites.

RNA editing, a key regulatory mechanism in the expression of mitochondrial and chloroplast genes in eukaryotes, involves the specific modification of transcripts to maintain the functional integrity of organelles (Mower, 2005; Bi et al., 2020). In this study, the PREP-Mt algorithm was used to systematically analyze the mitochondrial genome of *Camellia sinensis* cv. ‘Baihaozao’, identifying 159 C-to-U RNA editing sites across 25 protein-coding genes (PCGs) (**Figure 3**). These findings align with previous reports on the mitochondrial genome of *Phaseolus vulgaris* (486 sites) and the chloroplast genome of *Ginkgo biloba* (255 sites), supporting the dominant role of C-to-U editing in plant organelles (He et al., 2016; Bi et al., 2020).

This study identified 19 RNA editing sites in the *cox1* gene, significantly higher than the single editing site observed in the *rpl2* and *rps4* genes. This phenomenon is consistent with the general pattern of high editing frequencies observed in genes associated with mitochondrial complex I (Zhang et al., 2022b). Notably, a similar high number of editing sites in the *cox1* gene has not been widely reported in other species, suggesting that this feature may be specific to mitochondrial genomes in the genus *Camellia* (Cuenca et al., 2010; Zhang et al., 2022b).Codon position analysis revealed that editing events were predominantly located at the first (32%) and second (58%) bases, with markedly fewer at the third position (9%). This contrasts sharply with the complete absence of third-position editing in *Ilex metabaptista* (Aquifoliaceae), suggesting that different plant lineages may have evolved distinct strategies for editing regulation (Yura et al., 2008).

From a molecular mechanism perspective, 96% of the editing events corresponded to C-to-T changes at the DNA level (i.e., C-to-U editing at the RNA level), consistent with observations in model plants such as *Arabidopsis thaliana* and rice (Cummings and Myers, 2004; Zheng et al., 2020). Notably, leucine (43%) emerged as the most frequently produced amino acid following editing, potentially reflecting its critical role in transmembrane protein domains (He et al., 2016). Among the amino acid changes induced by RNA editing, 49% involve a transition from hydrophilic to hydrophobic residues. This hydrophobicity-biased trend aligns with the hypothesis proposed in studies on Ginkgo chloroplasts that suggests “enhanced protein structural stability” as a potential outcome (Daley et al., 2002). Although functional optimization of membrane proteins may be an underlying driving factor, the specific mechanisms remain to be further validated. Additionally, 42% of the sites retain their original hydrophobicity, while only 7% undergo a reverse transition (**Table 2**), implying that RNA editing may contribute to the adaptive regulation of the mitochondrial energy metabolism system through multiple mechanisms. It is worth noting that the analysis of RNA editing sites in this study is based on computational prediction (in silico) and has yet to be experimentally validated using transcriptomic approaches such as RNA-Seq or RT-PCR. Recent studies have demonstrated that mitochondrial editing sites in tea plants can be experimentally confirmed through PCR amplification and sequencing techniques (e.g., Zhang et al. identified 139 mitochondrial editing sites) (Zhang et al., 2022b). Future research will integrate transcriptomic data and molecular experiments to further validate the biological functions and regulatory mechanisms of RNA editing sites.

**Table 2:** Prediction of RNA editing sites.

Type	RNA-editing	Number	Percentage
hydrophilic-hydrophilic	CAC (H) => TAC (Y)	9	
	CAT (H) => TAT (Y)	18	
	CGC (R) => TGC (C)	12	
	CGT (R) => TGT (C)	30	
	total	69	12.80%
hydrophilic-hydrophobic	ACA (T) => ATA (I)	4	
	ACC (T) => ATC (I)	2	
	ACG (T) => ATG (M)	7	
	ACT (T) => ATT (I)	4	
	CGG (R) => TGG (W)	32	
	TCA (S) => TTA (L)	77	
	TCC (S) => TTC (F)	37	
	TCG (S) => TTG (L)	52	
	TCT (S) => TTT (F)	47	
	total	262	48.61%
hydrophilic-stop	CAG (Q) => TAG (X)	1	
	CGA (R) => TGA (X)	3	
	total	4	0.74%
hydrophobic-hydrophilic	CCA (P) => TCA (S)	8	
	CCC (P) => TCC (S)	9	
	CCG (P) => TCG (S)	3	
	CCT (P) => TCT (S)	20	
	total	40	7.42%
hydrophobic-hydrophobic	CCA (P) => CTA (L)	49	
	CCC (P) => CTC (L)	9	
	CCC (P) => TTC (F)	6	
	CCG (P) => CTG (L)	36	
	CCT (P) => CTT (L)	22	
	CCT (P) => TTT (F)	14	
	CTC (L) => TTC (F)	5	
	CTT (L) => TTT (F)	13	
	GCC (A) => GTC (V)	1	
	GCG (A) => GTG (V)	7	
	GCT (A) => GTT (V)	2	
	total	164	30.43%
	All	539	100%

### 3.3 Codon usage bias analysis

**Figure 4:**
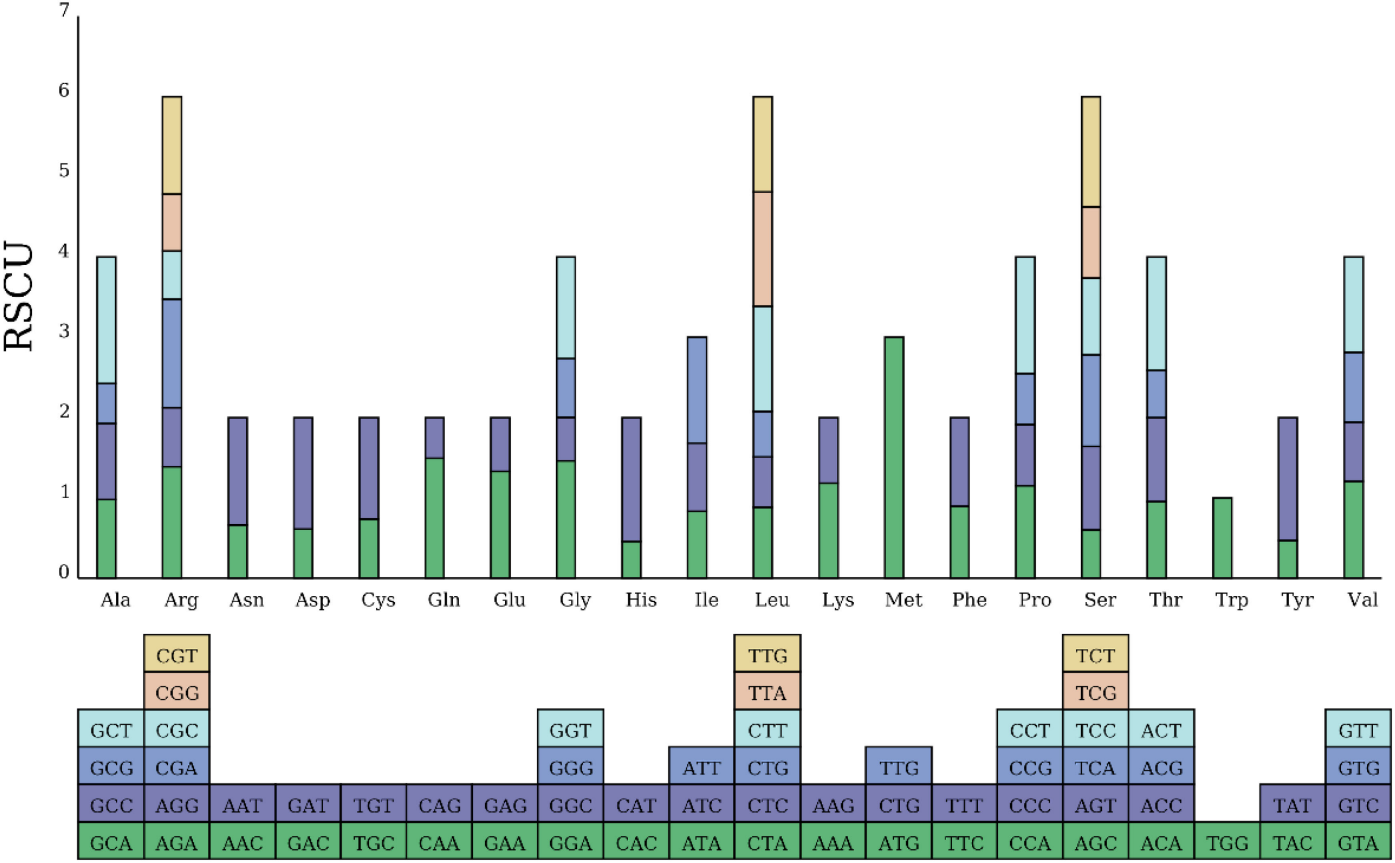
RSCU in the *Camellia sinensis* cv. ‘Baihaozao’ Mitochondrial Genome. The blocks below represent all codons encoding each amino acid, while the height of the bars above indicates the total RSCU values for all codons.

RSCU (Relative Synonymous Codon Usage) is a critical metric for assessing codon usage bias, and its calculation method and biological significance have been widely applied in genomic studies across multiple species (Qian et al., 2014; Yu et al., 2023). In this study, the mitochondrial genome of *Camellia sinensis* cv. ‘Baihaozao’ contains a total of 10,674 codons across 38 protein-coding genes. Among these, the aliphatic amino acid leucine (Leu) is the most frequently used (1,093 occurrences, accounting for 10%), followed by serine (Ser, 999 occurrences, 9%) and arginine (Arg, 739 occurrences, 7%), while tryptophan (Trp) exhibits the lowest usage frequency (157 occurrences, 1%) (**Figure 4**; **Supplementary Table 5**). This distribution pattern is consistent with the amino acid usage preferences commonly observed in mitochondrial genomes, such as in the stonefly (*Kamimuria wangi*) and the land snail (*Cernuella virgata*), where Leu and Ser also show high expression trends (Qian et al., 2014; Lin et al., 2016).

At the level of codon selection, there are 31 codons with RSCU > 1 in the mitochondrial genome of *Camellia sinensis* cv. ‘Baihaozao’. Among them, the start codon AUG (methionine) exhibits the highest RSCU value (3.0), reflecting its conserved role as a translation initiation site and the strong selection pressure it undergoes (Sau et al., 2005; Yu et al., 2023). This study identified high-frequency codons UUU (371 occurrences, 3%), AUU (358 occurrences, 3%), and UUC (300 occurrences, 3%) in the mitochondrial genome. The preference for such codons may be associated with nucleotide compositional bias driven by the high AT content of the genome (Sun et al., 2015; Li et al., 2019), or it may reflect selective pressures related to translational efficiency or evolutionary processes (Bulmer, 1991; Hershberg and Petrov, 2008). Notably, the corresponding amino acids, such as phenylalanine (Phe) and isoleucine (Ile), are involved in mitochondrial energy metabolism pathways. However, the potential link between codon enrichment and metabolic function requires further experimental validation (Hershberg and Petrov, 2008; Sun et al., 2015). Notably, this preference for A/T-rich codons has also been reported in the mitochondrial genomes of adenoviruses (FAdV) and lepidopteran insects (*Apostictopterus fuliginosus*), suggesting that codon selection in mitochondrial genomes may be driven by both genomic adenine and thymine (AT) content and translation efficiency (Han et al., 2018; Niczyporuk, 2018).

Compared to other plant mitochondrial systems, the codon usage pattern of *Camellia sinensis* cv. ‘Baihaozao’ exhibits species-specific characteristics. For example, in creeping bentgrass (*Agrostis stolonifera*), the RSCU value of the alanine (Ala) codon GCU is significantly higher than other synonymous codons (Li et al., 2023b), whereas in *Camellia sinensis* cv. ‘Baihaozao’ the leucine codon UUA is the dominant type. This difference may reflect the varying adaptive functional requirements of mitochondrial genomes during the evolutionary process among different species (Wang et al., 2019).

### 3.4 Repetitive sequence characteristics of the mitochondrial genome in *Camellia sinensis* cv. ‘Baihaozao’

The systematic analysis of repetitive sequences in plant mitochondrial genomes is of great significance for understanding their structural dynamics and evolutionary mechanisms. In this study, a comprehensive analysis revealed that the mitochondrial genome of *Camellia sinensis* cv. ‘Baihaozao’ contains three types of repetitive sequences: SSRs, tandem repeats, and dispersed repeats. This classification aligns with the classical definitions of repetitive sequences in plant organellar genomes. Specifically, SSRs, as a specialized type of tandem repeats, offer unique advantages in the development of genetic markers, while dispersed repeats significantly influence genome conformation by mediating homologous recombination (Zhu et al., 2024).

**Figure 5:**
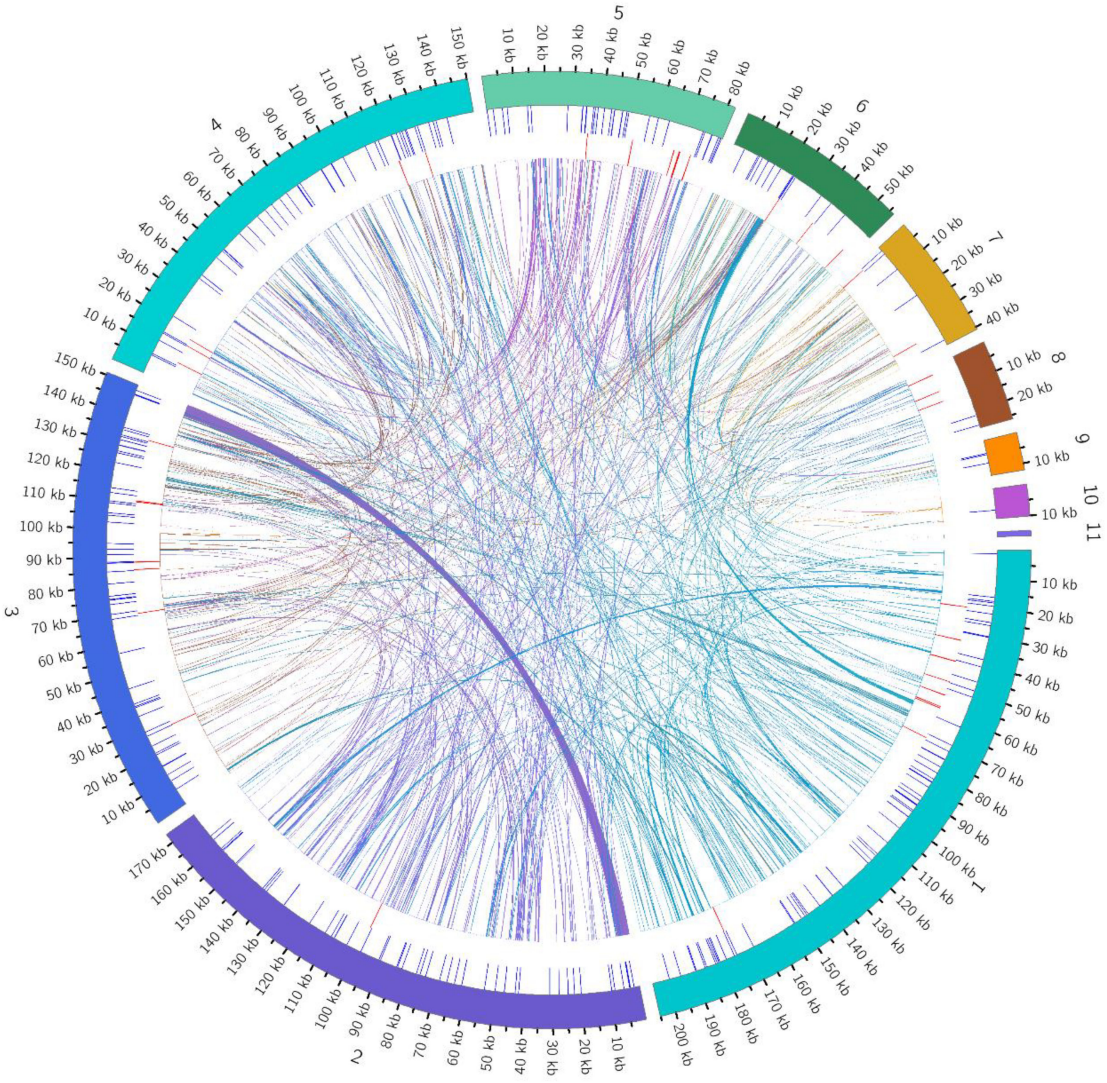
Distribution of repetitive sequences in the mitochondrial genome. The outermost circle represents the mitochondrial genome sequence, followed inward by simple sequence repeats, tandem repeats, and dispersed repeats.

Quantitative analysis detected a total of 1,032 repetitive sequences in the mitochondrial genome of *Camellia sinensis* cv. ‘Baihaozao’, of which SSRs (264 sequences) and tandem repeats (39 sequences) accounted for 26% and 4%, respectively, whereas dispersed repeats dominated with 729 sequences (71%) (**Figure 5**; **Supplementary Table 6**). This distribution pattern is similar to that of the mitochondrial genome in *Prunus armeniaca* (Rosaceae), where dispersed repeats account for 68%, suggesting that this type of repeat plays a widespread role in mitochondrial genome expansion in plants. Notably, the total length of dispersed repeats in *Camellia sinensis* cv. ‘Baihaozao’ reached 55,543 bp, comprising 6% of the genome, which is significantly lower than in *Selenicereus monacanthus* (85%, Solanaceae) (Lu et al., 2023), but higher than in common bean (*Phaseolus vulgaris*, 9%), reflecting substantial interspecies differences in the accumulation of repetitive sequences.

Subtype analysis of dispersed repeats revealed that forward repeats (333 sequences, 46%) and palindromic repeats (396 sequences, 54%) are the predominant types (**Supplementary Table 7**). These results are consistent with the general trend observed in plant mitochondrial genomes, where forward and palindromic repeats dominate. The presence of the largest forward repeat (890 bp) and palindromic repeat (4,713 bp) suggests their potential roles in homologous recombination, contributing to the formation of subgenomic linear molecules. This phenomenon has been experimentally validated in the multichromosomal structure of potato (*Solanum tuberosum*, Solanaceae). Additionally, dispersed repeats in the 30–40 bp length range were the most abundant (55%–70%), consistent with the observed length threshold (≥28 bp) in this study, indicating its universality in the analysis of plant mitochondrial repetitive sequences.

The analysis of tandem repeats (satellite DNA) showed that the 39 tandem repeats in *Camellia sinensis* cv. ‘Baihaozao’ ranged in length from 12 to 54 bp, with a match percentage >70%. Their chromosomal distribution exhibited significant heterogeneity: *chr1* and *chr5* each contained nine tandem repeats, while *chr9–11* were entirely devoid of such repeats (**Supplementary Table 8**). This uneven distribution pattern is consistent with the mitochondrial genome of *Cymbidium ensifolium* (Orchidaceae), where tandem repeats are enriched in *chr2* and *chr7* but absent in *chr13* (Shen et al., 2024). A comparative analysis revealed that the number of tandem repeats in *Camellia sinensis* cv. ‘Baihaozao’ is significantly higher than in common bean (7 repeats) but lower than in hawthorn (*Crataegus pinnatifida*, 22 repeats) (Zhu et al., 2024), indicating high plasticity of this repeat type among species. Notably, tandem repeats located in rRNA coding regions (e.g., the *rrnL* gene in common bean) may influence transcriptional regulation. However, no tandem repeats were found in the coding regions of *Camellia sinensis* cv. ‘Baihaozao’, suggesting that their function may primarily be associated with maintaining the structural integrity of non-coding regions.

### 3.5 Phylogenetic inference

**Figure 6:**
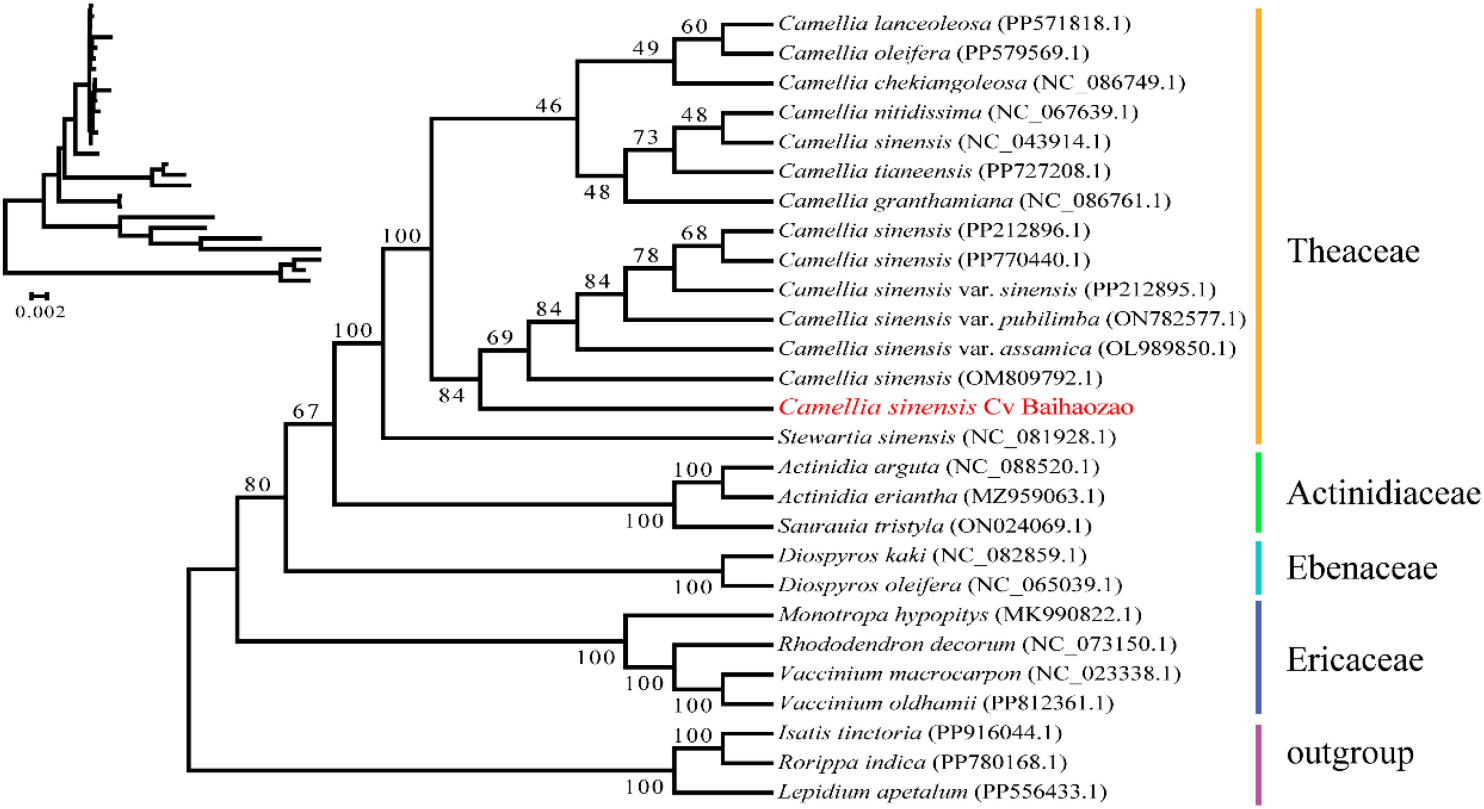
Phylogenetic relationships between *Camellia sinensis* cv. ‘Baihaozao’ and 26 other plant species.

The phylogenetic tree constructed based on 38 mitochondrial protein-coding genes (**Figure 6**) revealed that the 26 representative species were clustered into five monophyletic groups. Brassicaceae species (*Isatis tinctoria, Rorippa indica*, and *Lepidium apetalum*) were used as the outgroup, while the remaining species were grouped into Actinidiaceae, Ebenaceae, Ericaceae, and Theaceae. *Camellia sinensis* cv. ‘Baihaozao’ was clearly clustered within the Camellia clade of Theaceae, forming a highly supported monophyletic group (bootstrap value >90%) with *C. lanceoleosa, C. oleifera*, and other species, confirming the taxonomic reliability of its classification.

Notably, *Camellia sinensis* cv. ‘Baihaozao’ exhibited the closest genetic distances to the two major cultivated tea tree varieties, Chinese small-leaf tea (*C. sinensis* var. *sinensis*) and Assam large-leaf tea (*C. sinensis* var. *assamica*), suggesting a possible origin from a shared ancestral lineage. This finding is consistent with the divergence time of CSS and CSA estimated from whole-genome sequencing, which ranges from 0.38 to 1.54 Mya (Wei et al., 2017; Li et al., 2024b).

At the family level, significant evolutionary divergence was observed within Theaceae. Although *Camellia sinensis* cv. ‘Baihaozao’ and *Stewartia sinensis* (NC_081928.1) shared the same primary branch, they displayed clear genetic isolation (genetic distance >0.12), which may be attributed to distinct adaptive radiation events experienced by species of the two genera. Of particular interest, *Camellia and Polyspora* formed a sister group, with their divergence predating the split between *Camellia-Polyspora* and *Stewartia*. This supports the topology of the *Camellia-Polyspora* clade observed in comparative studies of chloroplast genomes (Chen et al., 2023a; Li et al., 2021).

Molecular evolutionary analysis showed that genes encoding components of oxidative phosphorylation complexes (e.g., *nad5* and *cox1*) in the mitochondrial genome of *Camellia sinensis* cv. ‘Baihaozao’ were under strong purifying selection (Ka/Ks < 0.5). In contrast, some genes involved in stress responses, such as *ccmB*, exhibited signals of positive selection (Ka/Ks > 1), suggesting specific functional optimization in response to environmental fluctuations (Chen et al., 2023b). This selection pattern is temporally and spatially associated with the whole-genome duplication events of secondary metabolic pathway genes in Camellia, such as those encoding theanine synthetase and flavonoid modification enzymes. These whole-genome duplication events have been shown to be key drivers in the evolution of tea quality traits (Wei et al., 2017; Li et al., 2024b).

### 3.6 Substitution rate analysis of Protein-Coding Genes

In molecular evolution studies, the ratio of Ka/Ks is widely used to assess the selective pressure acting on protein-coding genes. In this study, the nucleotide substitution model of Nei and Gojobori (1986) was applied in combination with multiple sequence alignments and Ka/Ks calculations using DnaSP v5 (Librado and Rozas, 2009). The evolutionary dynamics of 38 PCGs in the mitochondrial genome of *Camellia sinensis* cv. ‘Baihaozao’ were systematically compared with those of two species from the genus *Camellia* and six species from other genera.

**Figure 7:**
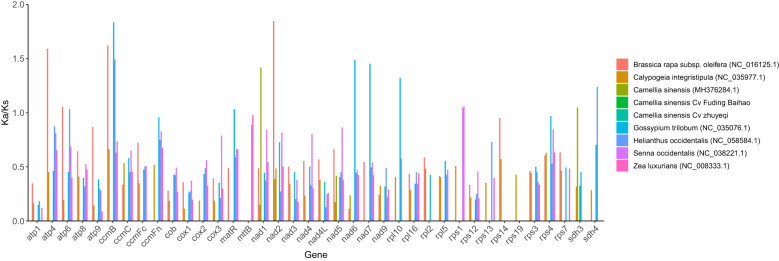
Boxplot of shared pairwise Ka/Ks values among mitochondrial genes in *Camellia sinensis* cv. ‘Baihaozao’.

The analysis indicates that among all PCGs, only 18 genes exhibit Ka/Ks values greater than 1, suggesting evidence of positive selection. Notably, the *atp9* gene shows the lowest ratio (0.07), while the *mttB* gene displays a markedly higher value of 3.48, implying that it may have undergone strong adaptive evolution (Li et al., 2024b). These findings align with the general trend of purifying selection observed in mitochondrial genomes while also revealing the distinct evolutionary trajectories of specific genes (Li et al., 2024b) (**Figure 7**; **Supplementary Table 9**). This pattern indicates that the mitochondrial PCGs of *Camellia* species have been subject to strong purifying selection over long-term evolution, maintaining high functional conservation.

It is worth noting that, although synonymous mutations have traditionally been considered neutral, recent studies suggest that synonymous sites may also be subject to selection due to their roles in splicing regulation, codon usage bias, or mRNA stability (Park et al., 2017; Savisaar and Hurst, 2024). For example, Savisaar and Hurst (2018) demonstrated that exon splicing regulation imposes strong selective pressure on synonymous sites (Savisaar and Hurst, 2024). However, the universally low Ka/Ks values observed in this study suggest that, even if non-neutral effects of synonymous substitutions exist, their overall impact on evolutionary rates is masked by the dominant effects of purifying selection (Moutinho and Walker, 2024).

### 3.7 Nucleotide diversity analysis

**Figure 8:**
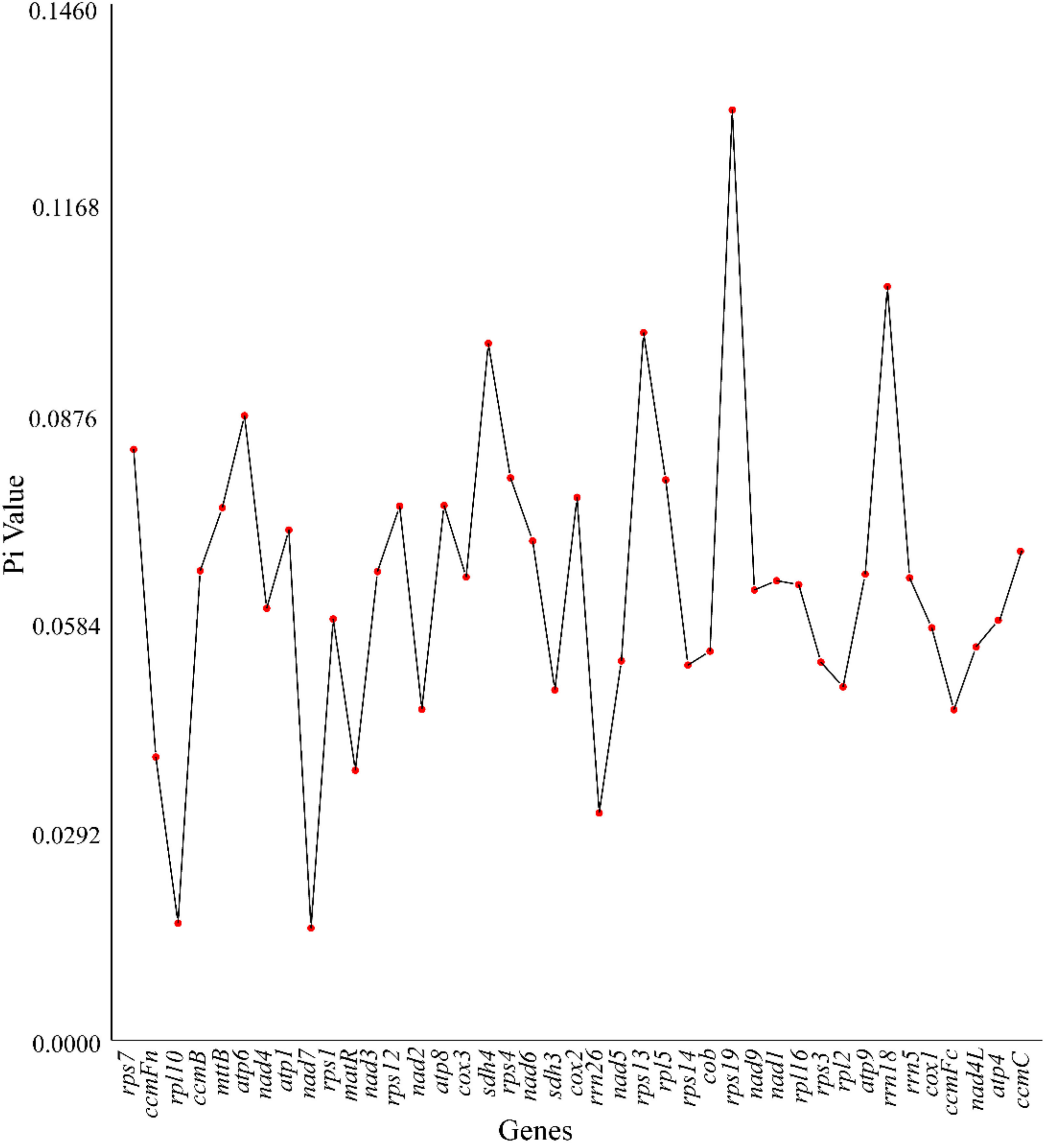
Pi among the mitochondrial genomes of nine selected *Camellia* species.

This study analyzed the nucleotide diversity (Pi) of 38 PCGs and three rRNA genes in the mitochondrial genome of *Camellia sinensis* cv. ‘Baihaozao’ to reveal its genetic variation characteristics (**Figure 8**; **Supplementary Table 10**). The results showed that the Pi values of the 41 genes ranged from 0.02 to 0.13, with *rps19* (Pi = 0.13) being the most variable PCG and *nad7* (Pi = 0.02) the most conserved (Liang et al., 2024). Notably, nine PCGs, including *rps13*, *sdh4*, and *atp6*, had Pi values exceeding 0.08, suggesting that these genes may be under relatively weak selective pressure or could serve as key candidate genes in adaptive evolution (Liang et al., 2024). Similar to chloroplast genome studies, this study also observed that ribosomal subunit-encoding genes (e.g., the *rps* and *rpl* families) exhibited higher variability, indicating functional convergence between mitochondrion and chloroplasts in genetic marker screening.

Among the rRNA genes, *rrn18* (Pi = 0.11) exhibited significantly higher variability than *rrn26* (Pi = 0.03) and *rrn5* (Pi = 0.07). This contrasts with the general pattern of high conservation observed in rRNA genes of chloroplast genomes in *Camellia* species (Yang et al., 2013; Li et al., 2021), possibly reflecting a distinct evolutionary trajectory of mitochondrial rRNA in species differentiation. For instance, the high variability of mitochondrial *rrn18* may be associated with its role in regulating translation fidelity or adaptation to energy metabolism, a hypothesis that requires further functional validation (Yang et al., 2013; Chen et al., 2021).

Cross-species comparisons revealed significant differences in the Pi distribution of *Camellia sinensis* cv. ‘Baihaozao’ PCGs compared to closely related species such as *Brassica rapa* (NC_016125.1), particularly in energy metabolism-related genes like *atp6* (Pi = 0.09) and *sdh4* (Pi = 0.08). These differences may be related to the unique secondary metabolic demands of tea plants, such as the specific requirements of the tea polyphenol synthesis pathway for mitochondrial redox balance. Moreover, genome-wide association studies suggest that structural variations, such as long terminal repeat retrotransposon expansions, may influence Pi distribution by introducing frameshift mutations and nonsynonymous substitutions, consistent with the distribution patterns of highly variable genes observed in this study.

It is worth noting that the IR regions in chloroplast genomes are typically highly conserved (average Pi = 0.0008) (Liang et al., 2024), whereas the structural heterogeneity of mitochondrial genomes may result in different variation patterns across regions. A recent pan-genomic analysis of 625 tea germplasms revealed a geographic gradient in the genetic diversity of cultivated populations, highlighting the need for future studies to integrate the cultivation history of *Camellia sinensis* cv. ‘Baihaozao’ to explore the population differentiation mechanisms underlying its mitochondrial Pi values. Furthermore, the high variability of marker genes such as *rps19* can be utilized in molecular-assisted breeding, and their potential as single nucleotide polymorphism markers requires experimental validation through population genetic studies.

### 3.8 Analysis of homologous fragments between mitochondrial and chloroplast genomes

**Figure 9:**
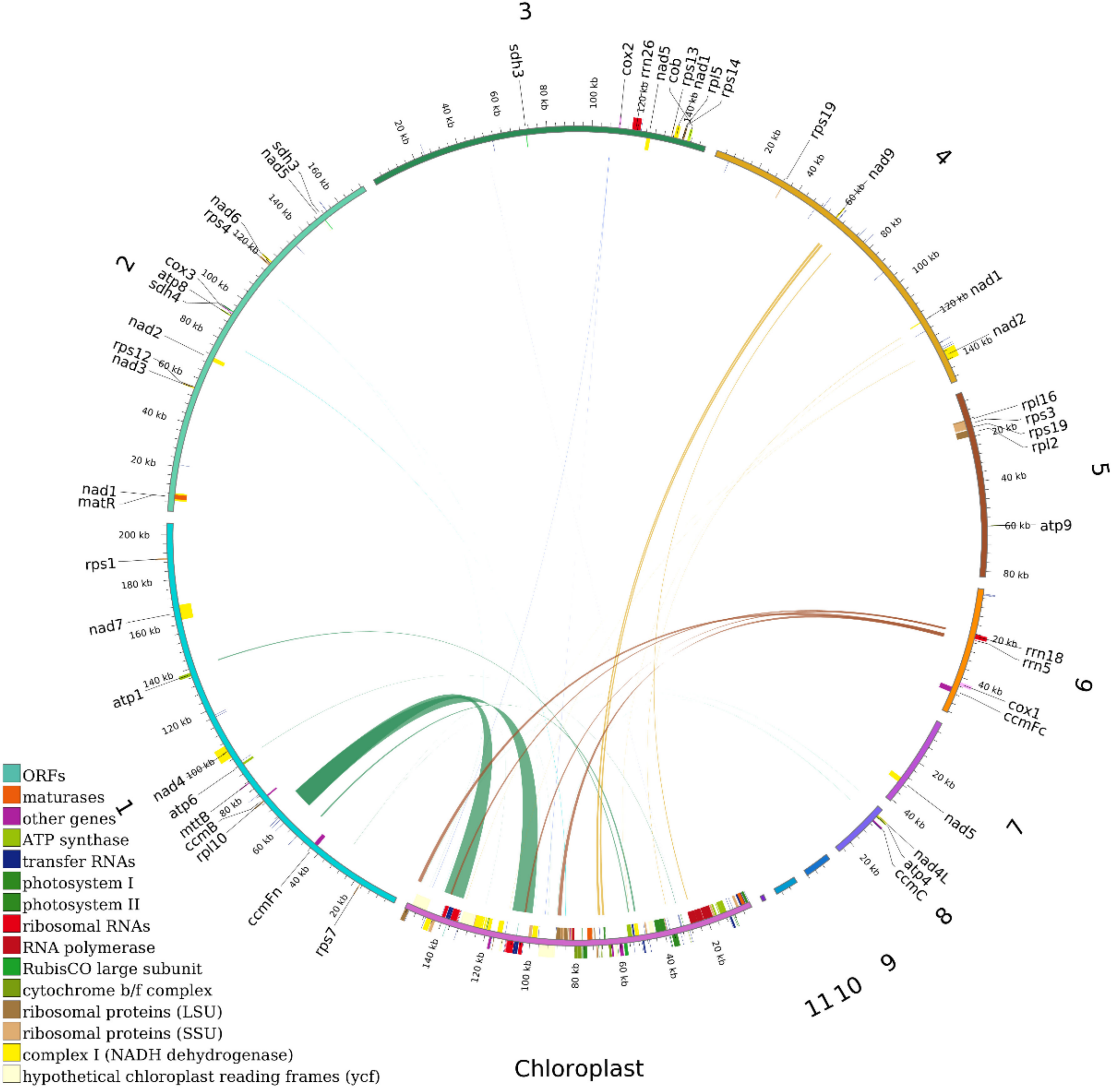
Distribution of homologous fragments between mitochondrial and chloroplast genomes in *Camellia sinensis* cv. ‘Baihaozao’. Chloroplast represents chloroplast sequences, while the others represent mitochondrial sequences. Genes belonging to the same complex are indicated by blocks of the same color. Blocks in the outer and inner rings represent genes on the forward and reverse strands, respectively. The connecting lines in the middle indicate homologous sequences.

This study conducted a systematic analysis of homologous fragments between the mitochondrial and chloroplast genomes of *Camellia sinensis* cv. ‘Baihaozao’. The mitochondrial genome (909,843 bp) was approximately 5.8 times larger than the chloroplast genome (157,052 bp; GC content: 37%), which exhibited the typical quadripartite structure of angiosperms (Large single-copy region: 86,586 bp; Small single-copy region: 18,227 bp; IRs: 52,162 bp) and encoded 133 functional genes. In this study, a total of 107 homologous sequence fragments were identified between the chloroplast and mitochondrial genomes, with their lengths ranging from 32 to 9,572 bp. Quantitative analysis revealed that the total length of homologous regions within the chloroplast genome was 27,578 bp, accounting for 17.56% of its total genome length. In contrast, the total length of homologous sequences within the mitochondrial genome was 14,733 bp, representing only 1.62% of its genome (**Figure 9**; **Supplementary Tables 11**, **12**). This result indicates that the transfer of chloroplast genome sequences to the mitochondrial genome exhibits a distinct directional preference, consistent with the general pattern observed in multiple species, where mitochondrial genomes integrate chloroplast sequences (Hao et al., 2022; Xie et al., 2024).

Annotation analysis revealed significant sequence homology between the chloroplast and mitochondrial genomes in the tea plant *Camellia sinensis* cv. ‘Baihaozao’. Specifically, 29 complete homologous fragments were identified in the chloroplast genome, involving genes such as *rrn23*, *rpl2*, *psbJ*, and *petL*, as well as ribosomal RNA genes, photosynthesis-related genes, and tRNA genes (e.g., *trnA-UGC*, *trnV-GAC*). In the mitochondrial genome, 14 complete homologous fragments were identified, including tRNA genes such as *trnV-GAC*, *trnA-TGC*, and *trnD-GTC* (**Supplementary Table 12**). These homologous fragments are likely the result of DNA transfer events between organelles, potentially mediated by mechanisms such as recombination facilitated by repeat sequences or random insertions (Liu et al., 2013).

The mechanism of inter-organellar gene transfer may be associated with recombination events mediated by repetitive sequences. Multiple forward and palindromic repeats were detected within the transferred fragments (**Figure 9**), resembling the mechanism observed in *Lithocarpus litseifolius*, where mitochondrial recombination hotspots facilitated the integration of chloroplast DNA (Qiu et al., 2024). Although the functions of most transferred genes (e.g., *rpl2*, *orf42*) remain unclear, some tRNA genes (e.g., *trnW-CCA*) retain functional activity in mitochondrial genomes, such as in *Stemona* species, suggesting that such transfers may compensate for the loss of mitochondrial tRNAs and help maintain the stability of the translation system (Niu et al., 2022; Ou et al., 2024). These findings provide new insights into the co-evolution of plant organellar genomes. Future research should integrate transcriptomic and proteomic data to validate the functional expression patterns of transferred genes (Zhang et al., 2012; Ou et al., 2024).

### 3.9 Synteny and evolutionary dynamics analysis of the mitochondrial genome

**Figure 10:**
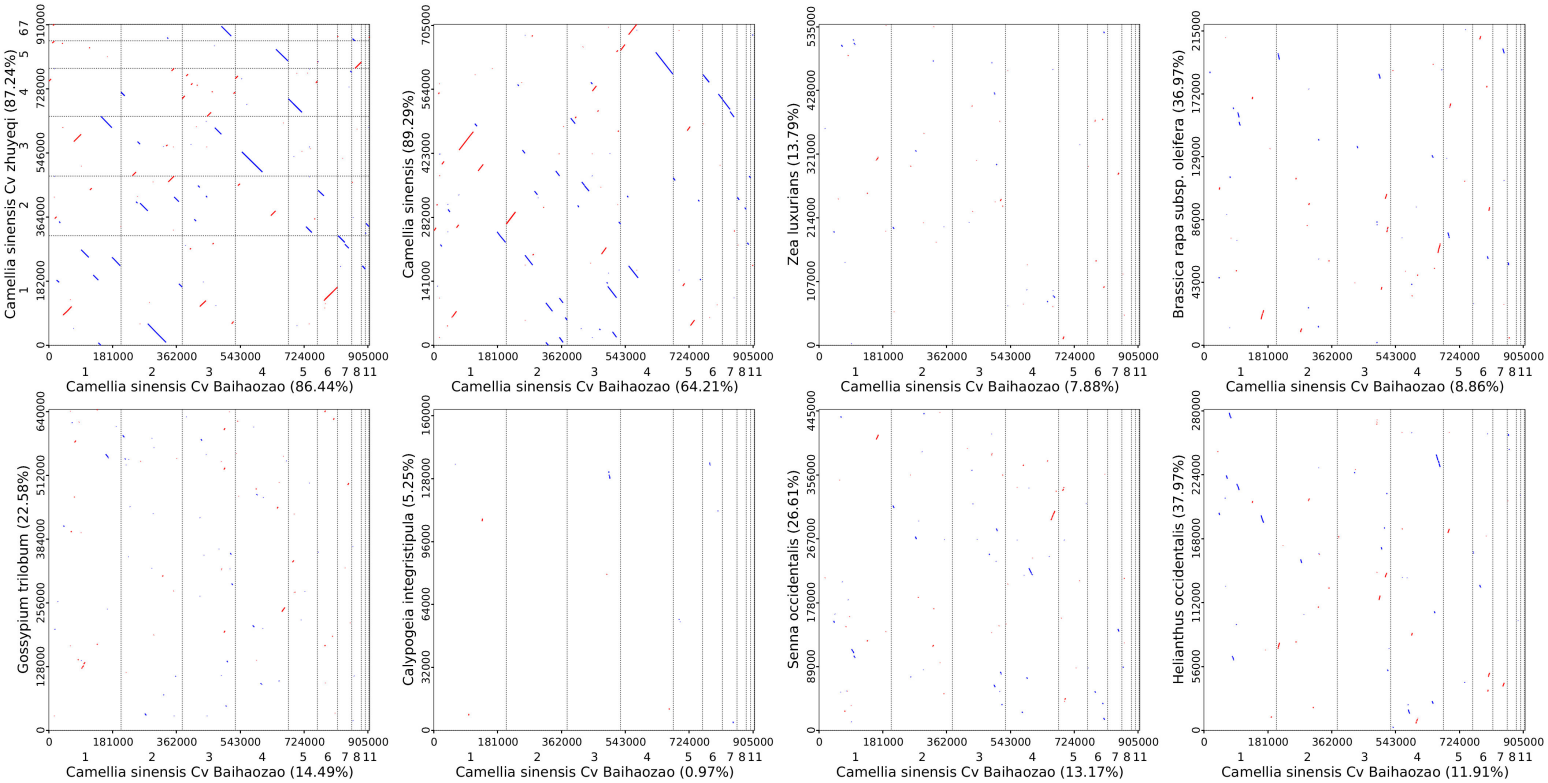
Homologous sequences among mitochondrial genomes of camellia species using *Camellia sinensis* cv. ‘Baihaozao’ as a Reference. The horizontal axis within each box represents the assembled sequence, while the vertical axis represents sequences from other species. The values in parentheses indicate the proportion of homologous sequences relative to the total genome size. Red lines within the boxes denote forward alignments, while blue lines represent reverse complementary alignments.

To investigate the structural evolution and phylogenetic signals of the mitochondrial genome of *Camellia sinensis* cv. ‘Baihaozao’, a whole-genome alignment strategy was employed to analyze its synteny with the mtDNAs of other species in Theaceae and representative species from other families (**Figure 10**). The results showed that *Camellia sinensis* cv. ‘Baihaozao’ exhibited the highest degree of synteny with ‘Zhuyeqi’ mtDNA (62% of aligned blocks in the forward orientation and 25% in the reverse complementary orientation), followed by *Camellia sinensis* (58% forward alignment). This indicates significant structural conservation of mitochondrial genomes among these species. This phenomenon is highly consistent with the phylogenetic results of chloroplast genomes, suggesting that organellar genomes may have undergone coordinated evolution during the divergence of *Camellia* species.

Further comparisons across families revealed that the number of homologous sequences among species within Theaceae (mean 1,428 ± 203) was significantly higher than that between Theaceae and species from other families, such as Rosaceae and Solanaceae (mean 327 ± 89) (**Supplementary Table 13**). This finding supports the “closely related species share a higher proportion of homologous sequences” hypothesis (Lai et al., 2022) and is consistent with the observed decline in synteny levels between genera in Solanaceae mitochondrial genome studies (Chen et al., 2024). Notably, although PCGs of mtDNA in Theaceae species exhibited high conservation (nucleotide similarity >99%), frequent structural rearrangements were observed in the non-coding regions, such as the dynamic expansion and contraction of IR boundaries (Liu et al., 2023). This feature resembles the evolutionary patterns of the mitochondrial genome in *Rubus chingii* (Rosaceae) (Shi et al., 2024).

## 4 Discussion

This study analyzed the multichromosomal structure, RNA editing characteristics, and adaptive evolutionary patterns of the mitochondrial genome in *Camellia sinensis* cv. ‘Baihaozao’ revealing its unique evolutionary dynamics and functional adaptations. These findings provide an important reference for mitochondrial genomics research in Theaceae. The 11 linear chromosomes of the mitochondrial genome of *Camellia sinensis* cv. ‘Baihaozao’ (total length: 909,843 bp) challenge the traditional understanding of a single circular structure in angiosperm mitochondria (Sloan, 2013). Its multichromosomal differentiation may result from recombination and fragmentation mediated by repeat sequences, with dispersed repeats accounting for 71% of the genome (Wynn and Christensen, 2019). Notably, ribosomal protein-coding genes (*rpl/rps*), which are commonly pseudogenized in Theaceae (Kubo and Arimura, 2010), remain fully functional and unmethylated in *Camellia sinensis* cv. ‘Baihaozao’. This preservation may be linked to its multichromosomal architecture, which enhances recombination flexibility and facilitates the elimination of deleterious mutations as part of an adaptive mechanism (Marechal and Brisson, 2010; Zhou et al., 2023). The mechanism underlying this multichromosomal differentiation may be attributed to frequent homologous recombination events or horizontal gene transfer. The high proportion of dispersed repeats (71%; 55,543 bp) likely mediates chromosomal fragmentation and rearrangement through recombination (Kurtz et al., 2001). Notably, variable genes (e.g., ribosomal subunit genes *rpl/rps*) often exhibit pseudogenization trends in Theaceae (Han et al., 2024), while *Camellia sinensis* cv. ‘Baihaozao’ retains their full functionality, suggesting its adaptive requirements for nucleo-mitochondrial interactions or energy metabolism. The poly-chromosomal structure may accelerate the purging of deleterious mutations by enhancing recombination flexibility (Murat et al., 2010; Betran et al., 2012; Peer et al., 2017), while simultaneously maintaining the functional integrity of essential genes (e.g., *rpl/rps*) through inter-chromosomal functional complementation (Terasaki Hart and Wang, 2024). This mechanism aligns closely with the rapid environmental response characteristics of *Camellia sinensis* cv. ‘Baihaozao’ in stress-resistance metabolic pathways, suggesting that the poly-chromosomal architecture is a significant driving force of adaptive evolution (Terasaki Hart and Wang, 2024).

The spatial heterogeneity of RNA editing sites (e.g., 19 sites in *cox1* vs. only 1 site in *rpl2/rps4*) reflects differences in functional constraints across genes. RNA editing predominantly occurred at the first and second codon positions (91%), which are closely associated with changes in amino acid properties. For instance, 49% of the editing sites resulted in hydrophilic-to-hydrophobic transitions, which may optimize protein conformation and enhance mitochondrial oxidative phosphorylation efficiency (Chen et al., 2012; Han et al., 2024). Additionally, the RNA editing site distribution pattern in *Camellia sinensis* cv. ‘Baihaozao’ is similar to that of other species such as *Rehmannia chingii* (Han et al., 2024). However, no reverse editing (U-to-C) was detected in *Camellia sinensis* cv. ‘Baihaozao’, indicating the conserved nature of the RNA editing mechanism in Theaceae (Zhang et al., 2020)). High-frequency editing genes (e.g., *cox1*) may dynamically regulate mitochondrial responses to environmental stress, consistent with findings of temperature-responsive RNA editing (e.g., dynamic editing in grapevine.

The high frequency of leucine (Leu, 10%) and serine (Ser, 9%) usage is closely associated with the functions of mitochondrial ATP synthase and the electron transport chain, and their codon preferences may be driven by translational efficiency or tRNA abundance (Lee et al., 2023). The highest RSCU value of the methionine initiation codon AUG (3.0) indicates its advantage in translation initiation efficiency. The preference for high-frequency codons such as UUU (Phe) and AUU (Ile) may be related to their critical roles in the hydrophobic cores of transmembrane proteins, further supporting the optimization of mitochondrial membrane system functionality (Han et al., 2024).

The dominance of dispersed repeats (71%) is consistent with the mitochondrial structural rearrangement patterns observed in species such as *Fargesia qinlingensis* (Wu et al., 2024), which likely facilitates genome plasticity through homologous recombination. The heterogeneous chromosomal distribution of tandem repeats (enrichment on *chr1*/*chr5* vs. absence on *chr9-11*) may reflect local recombination hotspots or selective pressures in functional regions. For example, *chr1* and *chr5* harbor multiple core genes (e.g., *atp* and *nad* clusters), where tandem repeats might stabilize transcriptional regulation (Benson, 1999). Additionally, the high abundance of SSRs (264) could serve as molecular markers, providing valuable resources for genetic diversity analyses in Theaceae (Lee et al., 2023).

In the assessment of evolutionary selection pressures on mitochondrial PCGs, Ka/Ks ratio analysis reveals that the majority of genes (Ka/Ks < 1) are under purifying selection, consistent with typical molecular evolutionary patterns (Zhang et al., 2022a, 2024). Notably, the *mttB* gene exhibits a significant signal of positive selection (Ka/Ks = 3.48), with its exceptionally high ratio potentially reflecting adaptive evolution in specific metabolic pathways, such as metal ion transport (Zhang et al., 2024b). In contrast, the *atp9* gene shows an extremely low ratio (0.07), indicating a highly conserved functional sequence. Pi analysis further reveals high variability in the ribosomal subunit gene *rps19* (Pi = 0.13), which may be associated with evolutionary demands for regulating translational accuracy. Conversely, the strong conservation of the *nad7* gene (Pi = 0.02) corresponds to its critical functional constraints in the assembly of mitochondrial complex I (Zhang et al., 2024b). This correlation between selection pressure and functional importance provides new insights into the evolutionary driving forces shaping mitochondrial genes.

The mitochondrial genome of *Camellia sinensis* cv. ‘Baihaozao’ contains 3% homologous fragments (29,491 bp) carrying 15 tRNA genes and 9 functional genes (e.g., *psbE*, *petL*), indicating frequent inter-organellar gene transfer events (Salmans et al., 2010; Han et al., 2024). These transferred fragments may enhance environmental adaptability by compensating for the loss of mitochondrial tRNAs or improving redox balance capabilities. Compared to the chloroplast gene transfer patterns observed in Oryza sativa (Notsu et al., 2002), the transferred genes in *Camellia sinensis* cv. ‘Baihaozao’ are more abundant, highlighting the unique role of Theaceae species in the co-evolution of organelles.

The multichromosomal architecture, dynamic RNA editing, and recombination mechanisms driven by repetitive sequences in the mitochondrial genome of *Camellia sinensis* cv. ‘Baihaozao’ collectively shaped its distinct evolutionary trajectory. These findings not only provide molecular markers for phylogenetic analyses of Theaceae but also offer new perspectives on the mechanisms underlying mitochondrial adaptive evolution. Future studies could integrate multi-omics data to further explore the dynamic regulation of RNA editing under environmental stress and the phenotypic associations of the multichromosomal structure.

## 5 Conclusions

This study presents the first complete sequencing and analysis of the mitochondrial genome of *Camellia sinensis* cv. ‘Baihaozao’. The genome consists of 11 linear chromosomes with a total length of 909,843 bp and a GC content of 46%, challenging the traditional view of a single circular mitochondrial structure. Among the 73 functional genes annotated in the genome, 24 core genes (from the *atp*, *nad*, *ccm*, and *cox* families) are highly conserved. Notably, ribosomal protein-coding genes (*rpl/rps*) have retained functional integrity despite the widespread pseudogenization observed in Theaceae plants. Their unmethylated state may be maintained by the recombination advantages conferred by the regulation of the poly-chromosomal structure. The genome displayed dynamic features, with dispersed repeats covering 71% (55,543 bp) of the sequence. Forward and palindromic repeats dominated and likely mediated frequent recombination events. RNA editing was unevenly distributed, with the *cox1* gene identified as a hotspot (19 sites). Over 90% of editing events occurred at the first and second codon positions, and 49% of these caused transitions from hydrophilic to hydrophobic amino acids, suggesting precise regulation of mitochondrial energy metabolism.

Adaptive evolutionary analysis indicates that the vast majority of PCGs have Ka/Ks values less than 1 (ranging from 0.07 to 0.78), with the *atp9* gene exhibiting the lowest ratio (0.07), consistent with the prevailing trend of purifying selection in mitochondrial genomes. Notably, the *mttB* gene displays a Ka/Ks value of 3.48, significantly higher than that of other genes, suggesting that it may have undergone positive selection to adapt to environmental pressures. Nucleotide diversity analysis revealed variability among functional genes: *rps19* showed the highest diversity (Pi = 0.13), while *nad7* was the most conserved (Pi = 0.02). Homologous fragments between the mitochondrial and chloroplast genomes (29,491 bp) carried 26 complete genes, 58% of which were tRNA genes, highlighting active inter-organellar gene transfer. Phylogenetic analysis clarified the evolutionary position of *C. sinensis* cv. ‘Baihaozao’ within Theaceae and provided insights into the plasticity of its mitochondrial genome, driven by its multichromosomal structure and dynamic repeats. This study establishes a genomic foundation for tea germplasm evaluation and molecular breeding. It also offers valuable insights into the evolutionary mechanisms of plant mitochondrial genomes.

## Data Availability

The datasets presented in this study can be found in online repositories. The names of the repository/repositories and accession number(s) can be found in the article/**Supplementary Material**.
